# Optimising Electroporation Condition for CRISPR/Cas-Mediated Knockout in Zona-Intact Buffalo Zygotes

**DOI:** 10.3390/ani14010134

**Published:** 2023-12-30

**Authors:** Meeti Punetha, Dharmendra Kumar, Sheetal Saini, Suman Chaudhary, Kamlesh Kumari Bajwa, Surabhi Sharma, Manu Mangal, Prem S. Yadav, Jonathan A. Green, Kristin Whitworth, Tirtha K. Datta

**Affiliations:** 1 Animal Physiology and Reproduction Division, ICAR-Central Institute for Research on Buffaloes, Hisar 125001, Haryana, India; 2 Division of Animal Sciences, University of Missouri, Columbia, MO 65211, USA

**Keywords:** RNP, electroporation, zygotes, buffalo, POU5F1

## Abstract

**Simple Summary:**

Genome editing is a well-known method for introducing targeted genetic alterations into livestock genomes. These changes must be transferable in the germline in order to be effective in animal breeding. Conventional methods of delivering CRISPR-Cas9 components, such as microinjection in the zygote or editing somatic cells followed by somatic cell nuclear transfer (SCNT), have demonstrated success in various species, including mice and certain domestic animals. However, these methods are often labour-intensive, technically demanding, and associated with variable efficiencies. Electroporation is a more recently described way of delivering Cas9 and sgRNAs into zygotes since it requires less expensive equipment than microinjection and takes less time. In the present study, we have developed an efficient method called CRISPR RNP electroporation of zygote (CRISPR-EP) to reduce mosaicism rates and increase biallelic mutations in buffalo. The developed easy and straightforward protocol for gene editing could serve as a useful method for studying the functional genomics of the buffalo embryos.

**Abstract:**

Somatic cell nuclear transfer or cytoplasm microinjection has widely been used to produce genome-edited farm animals; however, these methods have several drawbacks which reduce their efficiency. In the present study, we describe an easy adaptable approach for the introduction of mutations using CRISPR-Cas9 electroporation of zygote (CRISPR-EP) in buffalo. The goal of the study was to determine the optimal conditions for an experimental method in which the CRISPR/Cas9 system is introduced into in vitro-produced buffalo zygotes by electroporation. Electroporation was performed using different combinations of voltage, pulse and time, and we observed that the electroporation in buffalo zygote at 20 V/mm, 5 pulses, 3 msec at 10 h post insemination (hpi) resulted in increased membrane permeability and higher knockout efficiency without altering embryonic developmental potential. Using the above parameters, we targeted buffalo POU5F1 gene as a proof of concept and found no variations in embryonic developmental competence at cleavage or blastocyst formation rate between control, POU5F1-KO, and electroporated control (EC) embryos. To elucidate the effect of POU5F1-KO on other pluripotent genes, we determined the relative expression of SOX2, NANOG, and GATA2 in the control (POU5F1 intact) and POU5F1-KO-confirmed blastocyst. POU5F1-KO significantly (*p* ≤ 0.05) altered the expression of SOX2, NANOG, and GATA2 in blastocyst stage embryos. In conclusion, we standardized an easy and straightforward protocol CRISPR-EP method that could be served as a useful method for studying the functional genomics of buffalo embryos.

## 1. Introduction

The advent of genome editing (GE) has opened up new avenues for altering genomes. When compared to transgenic methods, new technologies like CRISPR-Cas9 have significantly increased the efficiency and precision of genome editing. Genes can be edited to generate a new product or to become non-functional by targeting specific regions of the genome. Genome editing technology emerged when the genetic breeding of animals was at a bottleneck stage. In the context of livestock genetics, particularly in economically significant species like buffaloes, the application of CRISPR-Cas9 technology holds immense potential for improving agricultural productivity, disease resistance, and overall livestock quality [1,2,3]. However, the successful implementation of CRISPR-Cas9-RNP-mediated genetic modifications in large animal species presents unique challenges [4]. One major hurdle is the efficient and accurate delivery of CRISPR-Cas9-RNP components into the developing embryos, or zygotes, of these animals.

Conventional methods for producing genome-edited animals are either delivering CRISPR-Cas9 components via microinjection in the zygote or editing somatic cells followed by somatic cell nuclear transfer (SCNT). Specific genetic alterations are performed on somatic cells (usually foetal fibroblasts), followed by the isolation of single-cell-derived colonies and cell screening to confirm the presence of desired genetic alteration. Typically, just one cell in a million carries the proper integration that results in a functional gene knockout. These positive cell clones need to be propagated after a long period of selection, which ultimately leads to a state of senescence. Nevertheless, an enucleated oocyte can be reconstituted using cells that have undergone proper integration [5,6,7]. SCNT is more technically challenging and time-consuming, and it is still an inefficient procedure with a low development rate; only less than 10% of the embryos transferred to recipients result in the birth of viable offspring [8,9,10,11]. The other common method involves pronuclear and/or cytoplasmic microinjection of Cas9 mRNA/sgRNA into pronuclear-stage embryos. The high cytoplasmic lipid content in large livestock like horse, cattle, and buffalo results in a dark cytoplasm that complicates pronuclear microinjection [12]. Moreover, this method is often labour-intensive, technically demanding, and associated with variable inefficiencies, making it less suitable for larger animals like buffaloes. Consequently, there is a growing need for alternative, simplified, and efficient delivery methods to facilitate successful genome editing in these economically important species.

Electroporation has emerged as a promising approach for the delivery of genetic material into cells, including zygotes [13,14,15,16,17]. It has been demonstrated that delivering CRISPR-Cas9-RNP of zygotes (CRISPR-EP) results in more rapid action, increasing mutagenesis efficiencies within the modified zygote [13]. This technique involves applying a brief electric pulse to cell membranes, creating temporary pores that allow the uptake of foreign molecules such as nucleic acids. Electroporation has been successfully utilized in various organisms and has shown potential for enhancing the efficiency of CRISPR-Cas9 delivery into embryos [18,19,20] Finally, CRISPR-EP is considerably less expensive as it requires only a stereomicroscope, an electroporator, and an electroporation cuvette, with technical simplicity and low-cost consumables [21].

Thus, as a proof of concept, we focused on the POU5F1 gene, which encodes the developmental regulator OCT4. POU5F1, a transcription factor and developmental control gene, regulates the development of mammalian embryos, cell lineage specification, and maintenance of germ cell pluripotency [22]. The specific role of POU5F1 in regulating embryonic development varies between species. POU5F1-null mice are able to produce blastocysts [23]; contrarily, bovine embryos deficient in POU5F1 experience developmental arrest around embryonic D5, limiting blastocysts formation during the initial lineage specification [24]. Furthermore, POU5F1-deficient porcine embryos reach the morula stage but fail to form the blastocoel [25]. Although POU5F1 is well known for its role in the development of mammalian embryos, the molecular mechanisms that regulate the first cell fate decisions in buffalo embryos are not well understood. The present study aims to address the challenges of efficient CRISPR-Cas9-RNP delivery in buffalo zygotes by investigating the feasibility and efficacy of a one-step electroporation method. This novel approach seeks to streamline the genetic modification process by combining the delivery of CRISPR-Cas9 components and the creation of DNA double-strand breaks in a single step. The central hypothesis is that one-step electroporation can enhance the precision and efficiency of CRISPR-Cas9-mediated POU5F1 mutations in buffalo zygotes, thereby assessing the role of POU5F1 in buffalo embryonic development using a loss-of-function approach.

## 2. Materials and Methods

### 2.1. Ethics Statement

All methods and experimental protocols were carried out in accordance with relevant safety guidelines and regulations of Institute Biosafety Committee and Institute Animal Ethics Committee, ICAR-CIRB, Hisar, India.

### 2.2. Guide RNA Designing and In Vitro Testing

The CRISPOR method was used to create a 20 nt buffalo POU5F1 gene-specific guide template [26] (Table 1). In order to create the sgRNA, the T7 promoter sequence was added to the guide template/forward primer. The guide template along with the Tracr Fragment and T7 Primer Mix were annealed using PCR to generate the gRNA DNA template. This, gRNA DNA template was in vitro transcribed to generate the sgRNA (GeneArt precision gRNA synthesis kit (#A29377, Invitrogen, Waltham, MA, USA). The sgRNA was further purified using the gRNA Clean Up Kit, GeneArt precision gRNA synthesis kit) and examined using a NanoDrop spectrophotometer.

### 2.3. Buffalo Fibroblasts Isolation and In Vitro Testing of sgRNA

The buffalo fibroblasts used in this study were isolated, cultured, characterised, and cryopreserved earlier by [27]. In brief, fibroblasts were isolated from skin-tissue biopsies taken from the underside of the tail, just above the anal region. At passages 2 and 3, the fibroblasts were cryopreserved using a slow-freezing method. At passage 2, a cryovial of buffalo fibroblasts was taken out from the liquid nitrogen tank and thawed at 37 °C in a beaker filled with water, where the ice crystals dissolved. Thawed cells were washed twice through centrifugation at 150× *g* with culture media containing DMEM/F12 (D8437, Sigma Aldrich, St. Louis, MO, USA) supplemented with 10% foetal bovine serum (F2442, Sigma Aldrich, USA) and 1% antibiotic-antimycotic solution (A5955, Sigma Aldrich, USA). Cell viability was determined using the Trypan blue exclusion dye. The viable cells were then plated at 0.3 × 10^6^ cells per well of 6-well plate in 2 mL culture media in a humidified CO2 (5%) incubator at 37 °C. When the cells were 70–80% confluent, 0.5 × 10^6^ cells were trypsinized and washed before being nucleofected with POU5F1 specific guides and true cut cas9 (100:100 ng/μL) in a 100 μL nucleofector solution (Amaxa 4D nucleofector kit for primary fibroblast cells; V4XP-2024) using the Lonza Nucleofector system programme EN-150. Cells were allowed to adhere in a 25 cm^2^ culture flask after transfection and were incubated in a CO_2_ incubator for 72 h. After 72 h, cells were harvested, and the knockout efficacy of guides was tested using a T7/E1 endonuclease assay (GeneArt™ Genomic Cleavage Detection Kit, A24372, Invitrogen, USA).

### 2.4. Oocyte Collection, In Vitro Maturation, In Vitro Fertilization and Zygote Collection

The buffalo ovaries were collected from an abattoir and transported to the laboratory within 4–6 h in sterile physiological saline containing antibiotic and antimycotic solution (A5955, Sigma Aldrich, USA) at 32 °C. The ovaries were aspirated, and the cumulus oocyte complex (COC) was collected with an 18 g needle filled with aspiration media (TCM-199 50 mL; L-glutamine 0.005 gm; BSA 0.33 gm; antibiotic 500 μL). Grade A and B COC were picked up under a stereo zoom microscope (Nikon, SMZ800N, El Segundo, CA, USA), washed in wash media (BO-Wash, 51002, IVF Bioscience, Falmouth, UK), and then placed in maturation media (BO-maturation, 71001, IVF Bioscience, UK) for 24 h in 5% CO_2_ at 38.5 °C. The matured oocytes were fertilized with 1 × 10^6^ spermatozoa/mL frozen-thawed buffalo sperm in BO-semen prep media (71003, IVF Bioscience, UK) and incubated for 8–10 h at 38.5 °C in 5% CO_2_. Following that, the cumulus cells were gently pipetted away from the presumed zygotes and used for electroporation.

### 2.5. RNP Complex Formation and Electroporation of Presumptive Zygotes and Culture

The probable buffalo zygotes were washed to remove COC and sperm 8–10 h post-insemination (hpi), followed by zone thinning with acid Tyrode’s solution (T1788, Sigma Aldrich, USA) for 10 s and washed twice with wash medium. Zygotes were electroporated in 50 μL of OptiMEM media using only Cas9 (100 ng/μL) (control) and POU5F1 gene specific sgRNA: Cas9 (100:100 ng/μL; mutated). Buffalo zygotes that had been thinned by zona pellucida (50–60 per electroporation reaction) were incubated in RNP complex for 10 min. The ECM 2001 BTX electrofusion machine (Harvard Bioscience, Holliston, MA, USA) was used to electroporate the CRISPR-Cas9-RNP in a 1 mm cuvette using square wave electroporation at 15 and 20 V/mm for 3 and 5 pulses conditions. The transfected zygotes were cleaned with wash media before being placed in in vitro culture media (BO-IVC media, 71005, IVF Bioscience, UK) to continue embryonic development. Cleavage and blastocyst rates were recorded on the second and eighth day after electroporation, respectively.

### 2.6. Extraction of Cell Lysate from Single Blastocyst and Genotyping for Validation of Knockout

At day 8 after zygote electroporation, a blastocyst stage embryo was retrieved to test knockout efficiency. Individual blastocysts were digested in 10 μL of Picopure DNA extraction buffer (KIT0103, Applied Biosystem; Waltham, MA, USA), then incubated at 68 °C for 3 h and inactivated at 95 °C for 15 min. The extracted genomic DNA was kept at −20 °C until further use. To test knockout efficiency, a targeted region of POU5F1 was amplified using PCR. In a 25 μL reaction containing 5 μL of genomic DNA, PCR was performed using primers spanning the target sequence (Table 1) under the following conditions: enzyme inactivation at 95 °C for 10 min, 40 cycles (denaturation at 95 °C for 30 s, annealing at 55 °C (TM) for 30 sec, extension at 72 °C for 30 s, followed by final extension at 72 °C for 7 min and holding at 4 °C. Using the Gene-jet PCR purification kit (K0701, Thermo Scientific, Vilnius, Lithuania), the PCR product from each blastocyst was cleaned. Low-EEO agarose special (MB002, Himedia, Mumbai, India) was used to make a 4% agarose gel after being dissolved in a 1× TAE solution. Each well was loaded with 2 μL of the PCR product, and electrophoresis was run for 90 min at 60 V. Gel documentation system (Chemidox XRS+, Bio-Rad, Hercules, CA, USA) was used to obtain gel images, and the appropriate product was then purified and sent for Sanger sequencing. By counting the bands, mosaic mutations were graded as described earlier [28]. Using Tracking of Indels by Decomposition (TIDE) software version 3.3.0 (https://tide-calculator.nki.nl, Netherlands Cancer Institute), which only required two Sanger sequencing runs from control cells and mutant cells, the knockout effectiveness of the sgRNAs and the mutations potentially produced were evaluated. Mutation and mosaic mutation analysis were done, and the results were documented.

### 2.7. Genotype Confirmation for Monoallelic and Biallelic Mutation

The blastocysts which were presumed to be mutated were further analysed for type of mutation using Guide-it genotype confirmation kit (632611, Takara Bio, San Jose, CA, USA). The PCR product of the edited blastocysts spanning the targeted sites (50 ng/μL) was incubated with guide-it recombinant Cas9 nuclease (500 ng/μL) at 37 °C for 5 min. In addition, the manufacturer’s instructions were followed when mixing the PCR product with Cas9 nuclease to create a reaction solution with BSA and 15× Cas9 reaction buffer. This solution was then incubated at 80 °C for 5 min and 37 °C for 1 h. The gel documentation system was used to further investigate the data.

### 2.8. Gene Expression

Reverse transcription was performed on cell lysate isolated from each individual blastocyst (control and mutant) using a simple 20× RT enzyme mix and 2× RT buffer (A25741, TaqMan gene expression cells to CT kit, Invitrogen, Vilnius, Lithunia). Finally, the Power SYBR PCR master mix (4368577, Applied Biosystem, USA) was used to perform the gene expression analysis for SOX2, NANOG, and GATA2 using specific primers (Table 1). A basic qPCR technique was used, with UDG activation at 50 °C for 2 min and initial denaturation at 95 °C for 2 min, followed by 40 cycles of denaturation at 95 °C for 5 s, annealing temperature (58 °C) for 15 s, and extension at 72 °C for 1 min. To obtain the cycle threshold (CT) values and amplification plots for all calculated parameters, the “SYBER green (with dissociation curve)” technique was employed. Amplification of a standardised dilution series was used to calculate the slope of real-time PCR efficiency. A negative control containing all components except the template was included in each sample to rule out primer dimer formation and non-specific amplification.

### 2.9. Statistical Analysis

The software SPSS 22 (IBM Corporation, Armonk, NY, USA) was used to determine the statistical significance of differences in different electroporation groups and relative gene expression using one-way analysis of variance, followed by Tukey’s honest significant difference (HSD) test as a multiple comparison test and Student’s *t*-test. GraphPad prism was used to generate the graphs (Graph Pad Prism 8.3.0). Differences were considered significant at *p* ≤ 0.05. All experimental data are shown as Mean ± SEM.

## 3. Results

### 3.1. Selection of Functional POU5F1 Guide RNAs

**Figure 1:**
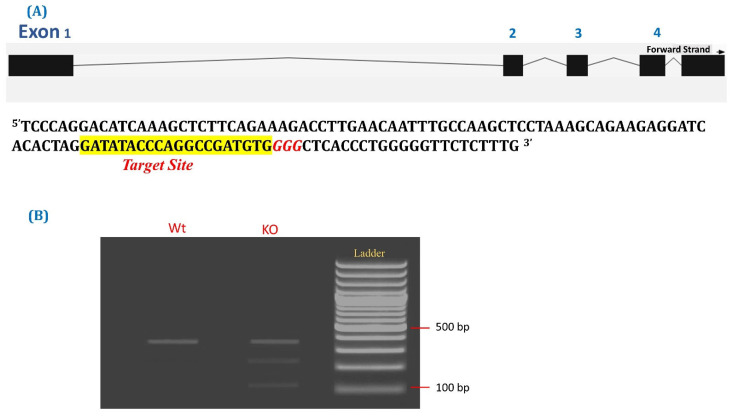
(**A**) Representation of buffalo POU5F1 gene structure and the genomic target site and guide designed to target POU5F1 exon 2. (**B**) Analysis of surveyor digestion showing that guide RNA is functional in vitro, lane Wt, control cell with enzyme; 2 KO, transfected cell with enzyme, parent band at 349 kb; cleaved bands, one band at 247 kb and other at 102 kb that corresponds to CRISPR/Cas 9 gene knockout in buffalo fibroblasts.

We designed a crRNA targeting POU5F1 domain against exon 2 using CRISPOR algorithm (Figure 1A). In order to check the functionality of guide, sgRNA-cas9 RNP was nucleoporated in cultured buffalo fibroblasts. The indel generation was tested using T7/E1 endonuclease assay after 72 h of nucleoporation (Figure 1B). The designed guide facilitated double-stranded cleavage in exon 2 of the POU5F1 gene. The tested guide was then utilised to investigate the role of the POU5F1 gene in buffalo embryos.

### 3.2. Effect of Post-Insemination Time and Voltage on Buffalo Embryonic Development

Buffalo in vitro-generated IVF zygotes were divided into two treatment groups at 8 and 10 hpi and were electroporated with Cas9 using 20 or 15 V, 3 P, 3 ms. To analyse the effect of introducing Cas9 by electroporation on blastocyst development, the blastocyst production rates were compared on day 8 after IVF. The cleavage and blastocyst rates were found to be considerably (*p* ≤ 0.05) higher at 10 hpi when compared to 8 hpi (Table 2). The increase in voltage, however, had no effect on the cleavage or blastocyst rate. The time of electroporation, on the other hand, had an effect on the rate of development to the blastocyst stage.

### 3.3. Effect of Different Electroporation Conditions on Knockout Efficiency

**Figure 2:**
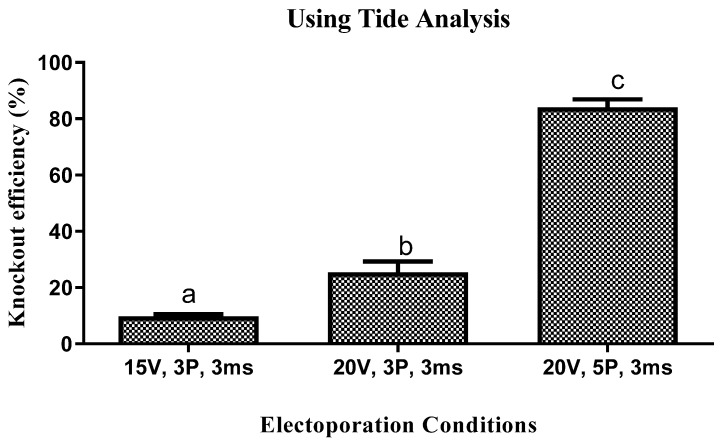
Knockout efficiency using TIDE analysis (*n* = 3) at different electroporation condition at 10 hpi. Each point in the line chart represents the mean ± SEM. Statistical significance (*p* ≤ 0.05) is denoted by different superscripts.

A representative proportion of blastocyst derived from different treatments of electroporation using POU5F1-targeted sgRNA and Cas9 at 10 hpi was analysed for knockout. The genomic lysate was isolated from individual blastocysts electroporated at 15 V and 20 V, 3 P for 3 ms and was Sanger sequenced. The knockout efficiency was evaluated using TIDE analysis (Supplementary Figure S1). At this situation, the knockout efficiency was 9.6 ± 0.72 and 25.3 ± 3.20% (Figure 2). However, when the pulse number was increased from 3 to 5, it considerably (*p* ≤ 0.05) increased the knockout efficiency to 84 ± 2.35 (Figure 2). As a result, for further experiment an electroporation setting of 20 V, 5 P for 3 ms was used.

### 3.4. Examination of Mutations and Mosaicism Produced by the Optimum Electroporation Condition (20 V, 5 P, 3 ms)

**Figure 3:**
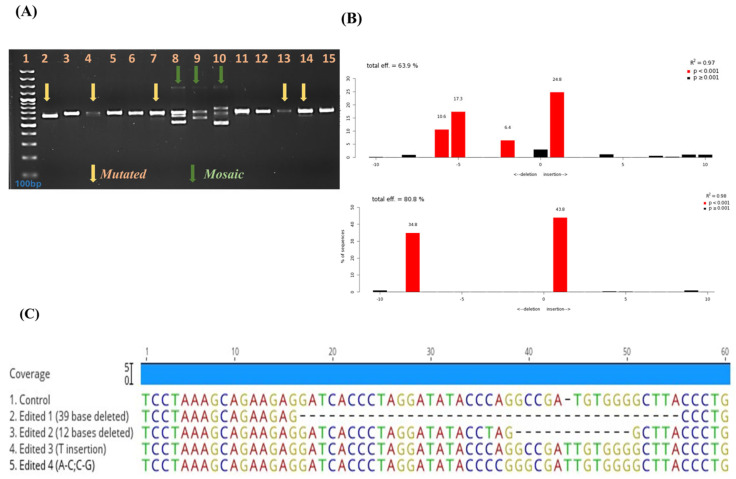
The PCR products of individual electroporated blastocyst spanning the targeted sites were run in 4% agarose gel to check the mutation and were further confirmed by sanger sequencing. Analysis of mosaic mutations was done by (**A**) gel electrophoresis by counting. Amplicons from WT or biallelic mutants produced single bands, and monoallelic mutants produced two distinct bands. Mosaic mutants were scored by counting the number of bands after 1 h of electrophoresis. (**B**) Using TIDE analysis, blastocysts with more than three alleles or two different alleles at rates of more than 25% were judged as exhibiting mosaicism (lane 8–10). (**C**) Alignment of sequences from representative blastocyst stage embryos targeted for POU5F1-KO using Geneious Prime software, Version 2023.2.1.

We examined genomic lysate samples of single blastocyst produced by electroporating buffalo zygote with a CRISPR-Cas9-RNP component at 20 V, 5 P, 3 ms in order to identify those containing genetic changes in the targeted POU5F1 gene. The PCR product of the genomic lysate of each blastocyst was directly sequenced using POU5F1 primers incorporating the target site (Table 1). In order to size-separate the DNA of representative wild-type (WT) and all various mutants, the PCR results were analysed on 4% agarose gels electrophoresis (Figure 3A). Amplicons from WT or biallelic mutants produced single bands after 1 h of electrophoresis that could not be distinguished in size. However, the biallelic mutant in lane 1 of Figure 3A produced a single band with a smaller amplicon size than the other bands. The PCR of monoallelic blastocysts resulted in two distinct bands and the mosaic mutant were scored by counting the number of bands (lanes 8–10). In order to test for mosaic mutation, TIDE analyses were also performed, and the results showed mosaic blastocysts with more than three alleles (Figure 3B) or the presence of two distinct alleles at rates higher than 25%. Sanger sequencing results for the POU5F1 targeted mutant embryo revealed 39 and 12 bp deletion in some instances, and one insertion at the targeted region in others (Figure 3C).

### 3.5. Genotype Identification Assay for Monoallelic and Biallelic Mutation

**Figure 4:**
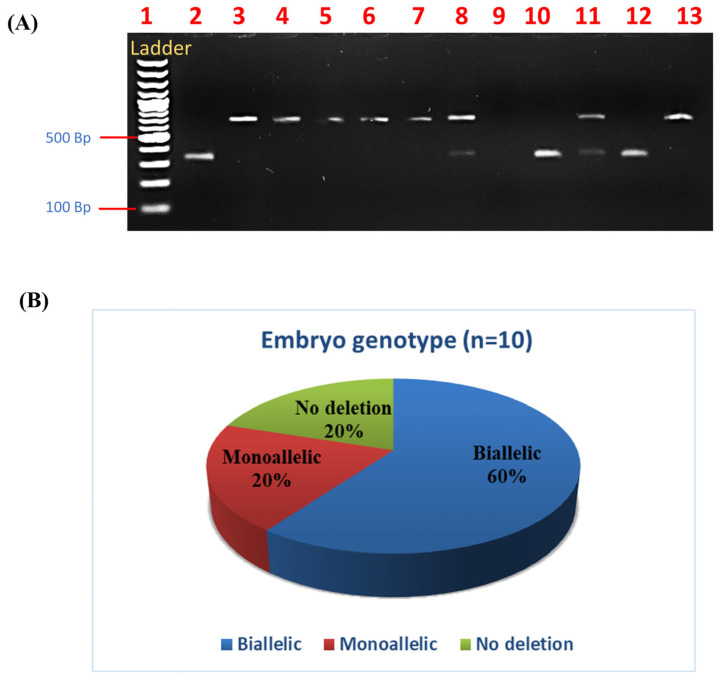
(**A**) Genotype identification assay products in 2% agarose gel using genotype confirmation kit, Lane 1: 100 bp ladder; lane 2: control (cleaved products size: 338 and 327; therefore, single band); lane 3–7 and 13: biallelic blastocyst (large fragment 665); lane 8 and 11: monoallelic mutation (large fragment 665 and cleaved band at 338 and 327); lane 10 and 12: wild-type blastocyst (merged cleaved products 338 and 327). Lane 9 zygote PCR product was insufficient to analyse (**B**) Genotyping of 10 electroporated blastocyst with RNP. Electroporation at 10 hpi at 20 V, 5 P, 3 ms resulted in significantly (*p* ≤ 0.05) higher biallelic knockout.

For further genotype confirmation of POU5F1-KO blastocyst, blastocysts were lysed and amplified using primers that included the target site listed in Table 1. On the generated PCR products, the cleavage process was conducted using the same POU5F1-specific sgRNA and Cas9 enzyme. The cleavage products were separated on an 2% agarose gel; for mutant cells, different banding patterns were present depending on the genotype. For biallelic mutants, the amplified allele was not cleaved, resulting in a single large band on the gel. For monoallelic mutants, only the amplified WT allele was cleaved, resulting in two small bands and one large uncut band, respectively. In the present study, the cleaved fragments were similar in size (338 and 327); therefore, they overlapped in the case of a wild blastocyst. Two bands (665 and an overlapped band at 338 and 327) were seen in monoallelic samples, while a single large band (665) was visible in biallelic samples (Figure 4A). The optimised electroporation conditions of 20 V, 5 P, and 3 ms at 10 hpi resulted in a significantly (*p* ≤ 0.05) higher biallelic knockout of 60% when compared to monoallelic (20%) and wild (20%) types (Figure 4B).

### 3.6. Effect of CRISPR-Cas9-RNP Induced POU5F1 KO on Blastocyst Formation and the Transcriptional Abundance of Pluripotency-Related Genes

**Figure 5:**
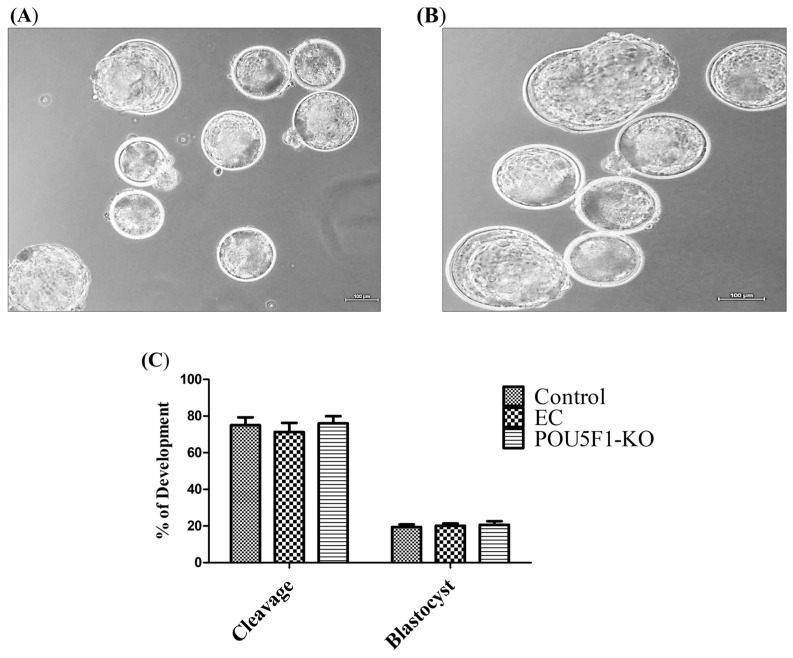
Embryo development following the electroporation of CRISPR/Cas9 targeting the POU5F1 was determined at the cleavage (D2) and blastocyst stages (D8) of oocytes fertilized with frozen-thawed bull semen. Representative images of blastocyst stage embryos of electroporated control (EC) (**A**) and POU5F1-KO (**B**). Cleavage and blastocyst rate on control (no electroporation) and after electroporation of buffalo zygotes with Cas9 (EC) and RNP-targeting POU5F1-KO (**C**). No significant difference between groups were observed (*p* ≥ 0.05).

**Figure 6:**
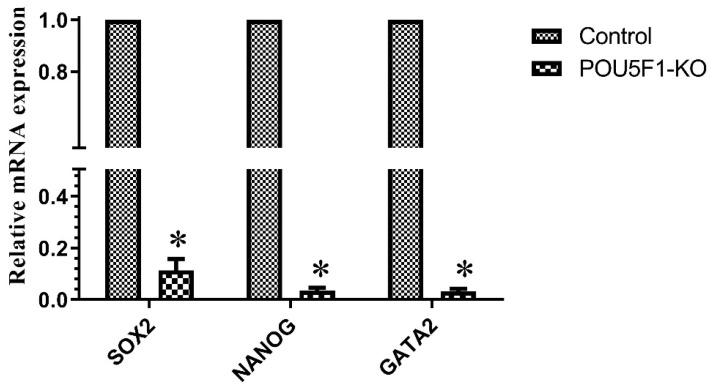
mRNA expression of SOX2, NANOG, and GATA2 in control and POU5F1-KO blastocyst. All values are shown as the mean ± SEM. * denote statistically different values (*p* ≤ 0.05).

The developmental potential of POU5F1-KO embryos was examined in order to understand the significance of embryonic POU5F1 for buffalo pre-implantation development. During blastocyst development and embryonic cleavage, no differences between control, POU5F1-targeted, and electroporated control embryos were observed (Figure 5A–C). Further, we also investigated the influence of POU5F1-KO on the pluripotent related genes; we measured the relative expression of SOX2, NANOG, and GATA2 in the control (POU5F1 intact) and confirmed POU5F1-KO blastocysts (confirmed by sequencing). For this, the TaqMan Gene Expression Cells-to-CT kit (AM1728, Thermo Fisher Scientific, Vilnius, Lithunia) was used in accordance with the manufacturer’s instructions to directly synthesize the complementary DNA from RNA in the cell lysate of confirmed edited and control blastocysts. In blastocyst-stage embryos, we found that POU5F1-KO significantly (*p* ≤ 0.05) changed the expression of SOX2, NANOG, and GATA2 (Figure 6).

## 4. Discussion

The present study aimed to optimize the CRISPR-EP method for buffalo zygotes. We have systematically investigated various parameters, including the voltage, pulse number, duration of pulses, and the embryonic stage at which the electroporation was performed. Traditional genome editing approaches often involve multiple steps, including the preparation of plasmids, transfection, and subsequent selection [29]. The current study bypasses these intricate steps by directly introducing the pre-formed CRISPR-Cas9-RNP complex into buffalo zygotes, streamlining the process significantly simple. This innovative technique has the potential to save time, reduce experimental variability, and increase the efficiency of genome editing procedures. However, CRISPR-EP is known to induce mosaic pattern in embryos [30]. So, in the present study, we also addressed the issue by considering the time window between fertilization and the first DNA replication [13] for the transfection of RNP. This study used CRISPR-EP to explore the role of genes in buffalo pre-implantation development and to create a way to decrease mosaicism rates while increasing genome editing efficiency.

In the current study, we showed, for the first time, that buffalo presumptive zygotes could be electroporated to produce INDEL mutations with remarkable efficiency. To lower the rate of mosaicism, the electroporation period was chosen to deliver the CRISPR component prior to the start of the DNA replication phase [31]. Prior research has [32] shown that chromosomal replication in in vitro produced bovine zygotes starts at the earliest time of 12 hpi and ends 18 h after the beginning of IVF. Therefore, in the current study, electroporation with only Cas9 was done at 10 and 8 hpi to check the developmental rate of zygotes. By shortening the oocyte-sperm co-incubation period at 8 hpi, the blastocyst and cleavage rates were considerably decreased [33,34,35] as compared to control and 10 hpi irrespective of electroporation condition, which may have been the result of the disturbance caused to the course of events in fertilization, which takes longer than 8 h. As a result, 10 hpi was determined to be the optimum time for electroporation in buffalo zygote for effective knockout utilising the CRISPR system. Overall, 50–60 presumed buffalo zygotes were electroporated with POU5F1 target-specific SgRNA/Cas9 at different electroporation conditions and were incubated for 8 days. The electroporation approach had no effect on the rate at which buffalo zygotes generated blastocysts, which was similar to a previous study in bovine [36,37]. At the blastocyst stage, the presumed POU5F1 knockout and control embryos were lysed, amplified, and sent for sequencing. Our settings, 20 V at 5 P and 3 ms, significantly improved knockout efficiency with high biallelic mutation and reduced mosaicism while having no negative effects on embryonic development; as a result, this voltage was chosen as the best one for electroporation.

In the current work, we also examined the effect of POU5F1-mutation on blastocyst development and cleavage rate. Early embryonic development and cell lineage specification depends on POU5F1, which encodes the developmental regulator OCT4 [38]. We observed that there was no significant difference in cleavage and blastocyst rate between POU5F1-KO and POU5F1 intact (control) embryos. Previous research found that knocking down POU5F1 in mouse embryos did not influence cleavage or blastocyst development rate but did reduce the expression of TE-specific genes in the ICM [38,39,40]. Simmet et al. (2022) [41] showed that POU5F1 is not required for blastocyst expansion and survival. In contrast, disruption of the POU5F1 locus in bovine hampered blastocyst development and demonstrated embryonic arrest at the morula stage [24]. The functional role of POU5F1 in embryonic development varies between species. Our findings show that POU5F1 is not required for the development of buffalo blastocysts. POU5F1 gene disruption affects blastulation but not embryo development up to the morula and blastocyst [24,42]. However, POU5F1-KO embryos considerably affect the expression of pluripotent genes SOX2, NANOG, and GATA2. POU5F1 (OCT4); SOX2 and NANOG are closely associated transcription factors that are necessary for both embryogenesis and the maintenance of pluripotency in cells. The OCT4 frequently interacts with the SRY-box-containing transcription factor SOX2 to control the expression of several target genes, such as important transcription factors like NANOG [43]. In a study, embryos with the POU5F1-KO mutation reached the blastocyst stage by day 8 but lost NANOG expression, while SOX2 and GATA6 were unaffected [41]. SOX2 expression was found to be downregulated when POU5F1 expression was regulated by RNA interference [44]. This demonstrates that POU5F1 positively regulates SOX2 and OCT4 alike. POU5F1 is required to maintain pluripotency in both murine and bovine embryos [23,45,46]. This is supported by the absence of NANOG, a pluripotency marker, in POU5F1-null bovine embryos [47,48]. It is interesting to note that transcription factor GATA2 expression in trophoblast cells was downregulated in POU5F1-null embryos in both human and mouse [49,50] trophoblast cells. Thus, we can say that the deletion of POU5F1 in buffaloes has no effect on first lineage differentiation but is required for pluripotency and NANOG expression.

## 5. Conclusions

In conclusion, this study successfully established a standardized protocol for introducing mutations in the POU5F1 gene of buffalo embryos using the CRISPR-EP method which represents a significant step forward in genome editing technology and could be a useful approach for analysing the functional genomics of buffalo embryos. The “one-step” aspect of the method simplifies the process, making it more accessible and efficient. CRISPR-RNP-induced POU5F1 mutation in buffalo embryos did not affect the blastocyst or cleavage rate. However, the loss of POU5F1 expression resulted in alterations in the ICM-specific pluripotency-related genes SOX and NANOG. Taken together, our study shows that POU5F1 is required for maintaining of pluripotency in the buffalo pre-implantation embryo throughout the second lineage differentiation; however, the validation of POU5F1 knockout on protein level was not done which is the limitation of the study to be addressed in future.

## Figures and Tables

**Table 1:** List of guides and primers sequence used in the study.

	Forward	Reverse
POU5F1 guide	TAATACGACTCACTATAGGATATACCCAGGCCGATGTG	TTCTAGCTCTAAAACCACATCGGCCTGGGTATATC
POU5F1 for T7E1 assay	GCCCTATGACTTGTGTGG	GGAAGGAAAACCCAGACTCC
POU5F1 for sequencing and genotype confirmation	CCCCCTTCCTAACCTGACAT	GTCGTTTGGCTGAACACCTT
SOX2	GTTCATCGACGAGGCCAA	CCCGGCAGTGTGTACTTATC
NANOG	GGGAAGGGTAATGAGTCCAA	AGCCTCCCTATCCCAGAAAA
GATA2	GCACAGCCGGACTAACTTAT	GGAATAGGAAGAGCGCATACA

**Table 2:** Details of developmental rates of one-cell-stage zygotes after electroporation using different conditions which included 10 and 8 hpi using 0, 15 and 20 V at 3 P for 3 msec.

Time of Electroporation Post Insemination (h)	Voltage (V/mm)	Zygotes (N)	Zygotes Kept for IVC, N	Cleaved, N (%)	Blastocyst, N (%)
10	0	468	468	355 (75.85 ± 1.84) ^a^	91 (25.86 ± 1.09) ^a^
	15	510	431	319 (74.2 ± 2.2) ^a^	90 (28.22 ± 2.49) ^a^
	20	479	435	298 (68.63 ± 3.22) ^a^	91 (30.57 ± 3.82) ^a^
8	0	295	295	136 (45.9 ± 1.89) ^b^	2 (1.67 ± 0.68) ^b^
	15	465	361	145 (40.03 ± 1.12) ^b^	3 (1.8 ± 0.84) ^b^
	20	459	420	157 (37.05 ± 2.40) ^b^	1 (0.49 ± 0.406) ^b^

Values with different superscripts within the same column differ significantly (*n* = 3; Mean ± SEM).

## Data Availability

The data presented in this study are available in article.

## Supplementary Materials

The following supporting information can be downloaded at: https://www.mdpi.com/article/10.3390/ani14010134/s1, Figure S1: Representative picture of TIDE analysis during different electroporation condition.
