# Supplementary material for: Optimising Electroporation Condition for CRISPR/Cas-Mediated Knockout in Zona-Intact Buffalo Zygotes
Source: Animals (Basel). 2023 Dec 30;14(1):134. doi: 10.3390/ani14010134 (PMC10778295; doi:10.3390/ani14010134)
Supplement: Supplementary file 1 [file animals-14-00134-s001.zip › animals-2761932-supplementary.pdf]

Supplementary file

Optimising Electroporation Condition for CRISPR/Cas-Mediated Knockout in Zona-Intact Buffalo Zygotes

Meeti Punetha [1,†], Dharmendra Kumar [1,*,†], Sheetal Saini [1], Suman Chaudhary [1], Kamlesh Kumari Bajwa [1], Surabhi Sharma [1], Manu Mangal [1], Prem S. Yadav [1,*], Jonathan A. Green [2], Kristin Whitworth [2] and Tirtha K. Datta [1]

[1] Animal Physiology and Reproduction Division, ICAR-Central Institute for Research on Buffaloes, Hisar 125001, Haryana, India

[2] Division of Animal Sciences, University of Missouri, Columbia, MO 65211, USA
* Correspondence: dharmendra.kumar@icar.gov.in (D.K.); psycirb@gmail.com (P.S.Y.)

† These authors contributed equally to this work.

Electroporated Blastocyst at **15V, 3P, 3ms**

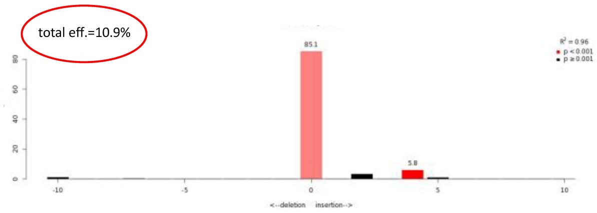

Electroporated Blastocyst at **20V, 3P, 3ms**

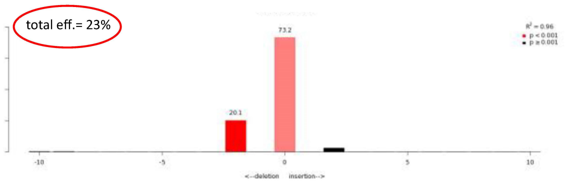

Electroporated Blastocyst at **20V, 5P, 3ms**

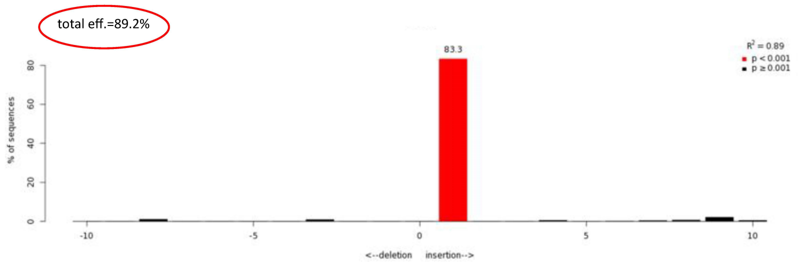

Figure S1: Representative picture of TIDE analysis during different electroporation condition.
